# Peptide-Based Treatment: A Promising Cancer Therapy

**DOI:** 10.1155/2015/761820

**Published:** 2015-10-19

**Authors:** Yu-Feng Xiao, Meng-Meng Jie, Bo-Sheng Li, Chang-Jiang Hu, Rui Xie, Bo Tang, Shi-Ming Yang

**Affiliations:** Department of Gastroenterology, Xinqiao Hospital, Third Military Medical University, Chongqing 400037, China

## Abstract

Many new therapies are currently being used to treat cancer. Among these new methods, chemotherapy based on peptides has been of great interest due to the unique advantages of peptides, such as a low molecular weight, the ability to specifically target tumor cells, and low toxicity in normal tissues. In treating cancer, peptide-based chemotherapy can be mainly divided into three types, peptide-alone therapy, peptide vaccines, and peptide-conjugated nanomaterials. Peptide-alone therapy may specifically enhance the immune system's response to kill tumor cells. Peptide-based vaccines have been used in advanced cancers to improve patients' overall survival. Additionally, the combination of peptides with nanomaterials expands the therapeutic ability of peptides to treat cancer by enhancing drug delivery and sensitivity. In this review, we mainly focus on the new advances in the application of peptides in treating cancer in recent years, including diagnosis, treatment, and prognosis.

## 1. Introduction

Peptides are molecules formed by combinations of amino acids linked by peptide bonds through the dehydration-condensation reaction. Peptides can be obtained conveniently from the products of proteolysis, direct synthesis by the body, or artificial synthesis [1]. Peptides play a large role in the treatment of diseases. Peptide-based therapy has been applied in various diseases, such as allergic diseases, infectious diseases, autoimmune diseases, fibrosis, and asthma. There are several advantages of peptides, such as easy availability and convenient purification and storage [2–4]. Peptide-based therapies have been tested in both in vitro and in vivo experimental models, and some may present promising outcomes. Larché demonstrated the basic mechanisms and feasibility of peptide therapy for allergic diseases [2]. Additionally, Iikuni et al. demonstrated encouraging efficacy using anti-DNA immunoglobulin peptide therapy against systemic lupus erythematosus (an autoimmune disease) in murine models [1]. Moreover, Oh and Lee reported the combined use of HMGB-1 box A peptide and S1PLyase siRNA in the treatment of acute lung injury [5]. Similarly, Nojiri et al. also certified that atrial natriuretic peptide (ANP) was significantly useful in inhibiting lipopolysaccharide-induced acute lung injury [6].

In addition, peptides also play an important role in cancer, including early diagnosis, prognostic predictors, and the treatment of cancer patients. Unlike other therapies, peptides show superiority due to their specificity. Recently, peptide-based therapy against cancer, such as peptide vaccines, has attracted increased attention [7]. Since sipuleucel-T was accepted by the US Food and Drug Administration (FDA) as the first standard peptide vaccine for prostate tumors, an increasing number of clinical trials have been conducted in many other cancer types, such as melanoma, glioblastoma, breast cancer, and gastric cancer [8]. However, the clinical response is considered limited and most of the current clinical trials showed limited efficacy [9]. Therefore, many novel methods, such as the combination with nanomaterials and chemotherapy, personalized peptide vaccination, and improved delivery systems, have been attempted in clinical trials and some may prolong the survival of cancer patients or result in tumor regression and show enhanced clinical efficacy.

In this view, we summarize the new progress of peptides in the application of cancer diagnosis (Table 1), prognostic predictors, and novel peptide therapies for cancer patients. We also discuss the prognosis and adverse effects of peptide vaccines in clinical trials.

## 2. Peptides and Colorectal Cancer

Colorectal cancer (CRC) is one of the most common cancers in the world, and it causes approximately 500,000 deaths worldwide per year, according to a recent report. Patients with metastatic CRC have a low 5-year survival rate, and early diagnosis of CRC leads to a better prognosis [19, 56]. At present, peptides also play an important role in CRC diagnosis. FITC-labeled peptide EPPT1 was linked to cationic polyacrylamide (CPAA) to form CPAA-783-EPPT1, which can target the cell transmembrane underglycosylated MUC-1 protein in the colorectal cell lines HT-29 and LS-174T [20]. In addition, in a recent clinical study, human neutrophil peptides 1–3 (HNP1–3) were reported to be present at high concentrations in CRC tissue, especially in Dukes' stages C and D [19]. Additionally, Comstock et al. also reported that a high concentration of serum C-peptide indicates a high risk of an adenoma in males [21]. These data suggested that peptides could be used as biomarkers for detecting CRC.

In addition to their function in the detection of CRC, peptides have also demonstrated their ability to treat CRC. Atrial natriuretic peptide (ANP), one of the cardiac and vascular derived peptide hormones, was reported to be a potential drug for CRC because it has antiproliferative effects in CRC cells [37]. Neovascularization is essential for tumor growth, and the neovasculature has been an attractive target for anticancer therapy. Li et al. reported that a peptide called TCP-1 could specifically target the blood vessels in tumor tissues [38]. Interestingly, they also found that TCP-1 could also deliver fluorescein and drugs for imaging detection and apoptosis in CRC, respectively [38]. Their findings suggested that TCP-1 could be a promising peptide for CRC therapy because it could carry anticancer drugs specifically to CRC tissue, without binding to normal tissue. In addition, Wang et al. also reported that F56 peptide conjugated nanoparticles loading vincristine (F56-VCR-NP) could target both primary lesions and the neovasculature of lung metastases, causing apoptosis of the neovasculature and necrosis of the CRC tissue [39].

Interestingly, peptides present in nondigestible fractions (NDF) of the common bean were reported to have an antiproliferative effect via increased p53 expression in a human CRC cell line [50]. In addition, researchers from the same team also found that the peptides in NDF from common beans could cause different gene expression in a human CRC cell line, which was related to cell death and survival, the cell cycle, cell proliferation, and so forth, leading to the induction of apoptosis and cell death. Their reports indicated that the role of peptides from the common bean could be used for anticancer treatment in CRC.

Additionally, the vaccine made by combined peptides has been well studied in treating CRC. Inoda et al. reported that the combined use of three peptides (Cep55/c10orf3_193(10), Cep55/c10orf3_402(11), and Cep55/c10orf3_283(12)) was effective in HLA-A24-positive CRC [51]. Recently, Hazama et al. reported a “peptide cocktail” treatment in CRC patients. In this study, researchers showed an improved median overall survival time in patients who received an injection of the “peptide cocktail” compared with the control group [57]. Then, in the following research, Hazama and colleagues found that the interleukin-6 level was increased due to the peptide vaccine, and it could also predict good prognosis in patients who accepted the peptide vaccine [52]. Similarly, Okuno et al. also reported that a 7-peptide cocktail vaccine with oral chemotherapy demonstrated an improved outcome in patients with metastatic CRC, as these subjects had a longer survival time compared with the control group [53].

From these data, we conclude that peptides could be used in detecting and treating CRC, and the combined use of peptides was most effective, including both peptides bound with a drug and the use of a “peptide cocktail” vaccine.

## 3. Peptides and Lung Cancer

Lung cancer is the leading cause of cancer-related mortality, and the 5-year survival remains low despite new therapies [58, 59]. The detection of lung cancer in early stages has attracted much attention in recent years. It has been many years since a peptide was first used as a predictor of lung cancer [60]. In recent years, new peptides have also demonstrated their ability in detecting lung cancer. Wang et al. reported that the linear peptide antigen derived from annexin A1 was higher in patients with non-small-cell lung cancer (NSCLC), compared with control subjects [22]. Zhang and colleagues also found that C-peptide in the serum was higher in patients with lung cancer, especially in the small cell lung cancer group, the stage III-IV group, and patients with lung cancer and diabetes [23]. Additionally, McGuire et al. found 11 novel peptides that specifically bind to a series of human NSCLC cell lines and are involved in a number of pathways, indicating that these peptides could be used as predictors for NSCLC [24]. Interestingly, peptides could also be used as carriers, because they have specific binding sites. Gniazdowska et al. reported that the vasopressin peptide conjugated with 99mTc (99mTc(NS3)(CN-AVP(an))) could be used as an ideal compound for imaging small cell lung cancer (SCLC) cells with its high stability and novel binding to the SCLC cell line H69 [61]. In addition, Hong et al. also found that 131I-anti-ProGRP(31–98)scFv, which can bind to progastrin-releasing peptide(31–98) (ProGRP(31–98)), had a high level of selective uptake by tumor tissues, but a low level in normal tissues, indicating that it could be used for SCLC radioimmunoimaging [62]. Impressively, de Costa et al. first found that peptides generated from variable antibodies were shared among lung cancer patients but not a control group [25], suggesting that these peptides could be novel biomarkers for screening lung cancer. Evidence also showed that the peptide HCBP-1 has exhibited specific binding to lung cancer stem cells, suggesting that this peptide may be used to identify lung cancer stem cells and as a drug carrier to lung cancer stem cells [26].

Peptides can also be used to treat lung cancer. Takahashi et al. reported that the dendritic cell vaccines pulsed with Wilms' tumor-1 peptide significantly improved the survival period of patients with advanced NSCLC [40]. Additionally, Kotsakis et al. also reported that the hTERT-targeting Vx-001 vaccine, which is a vaccine consisting of a TERT572Y optimized cryptic peptide that restricts target HLA-A^*∗*^0201, could induce strong immune responses and improve the clinical outcome of the majority of NSCLC HLA-A2 (+) patients [41]. Recently, Ahsa et al. also reported that the synthesized peptide Disruptin decreased the clonogenicity of EGFR-dependent cancer cells [42]. They also found that Disruptin could inhibit the microvessel density in lung cancer cell line H1975 xenografts [42], indicating that Disruptin could be a potential drug for EGFR positive cancer. Similarly, Sigalov also designed a ligand-independent peptide-based TREM-1 (triggering receptor expressed on myeloid cells-1) inhibitor to specifically silence TREM-1, and this peptide delayed tumor growth in xenograft models of human NSCLC [43].

Interestingly, peptides from natural sources were also able to treat lung cancer. The peptide fractions from high oleic acid soybean showed an inhibitory effect in cancer cells (including colon cancer, liver cancer, and lung cancer), and this effect was dose dependent [54]. Additionally, peptides from the venom of the Eastern green mamba have toxic effects against the human NSCLC cell line A549 [55].

With the development of nanotechnology, peptides that are conjugated with nanomaterials have exhibited a great potential in treating diseases, especially cancer. Chittasupho et al. reported that a synthetic compound (LFC131-DOX NPs), which contained a peptide (LFC131, an inhibitor of CXCR4), PLGA nanoparticles, and doxorubicin, could specifically bind to the human lung cancer cell line A549, indicating that LFC131-DOX NPs could be used as a drug delivery system in treating lung cancer [44]. In addition, Guan and colleagues also reported that TH10 peptide conjugated nanoparticles loading docetaxel (TH10-DTX-NP) showed therapeutic efficacy in inhibiting vascular pericytes in a mouse lung metastasis model, indicating that TH10-DTX-NP could be a potential drug for treating cancer [46]. Similarly, Wang et al. also reported that bradykinin-potentiating peptide (BPP) decorated chitosan nanoparticles could enhance vascular permeability in tumors, resulting in drug accumulation in tumors and prolonging survival [45].

## 4. Peptides and Pancreatic Cancer

Pancreatic cancer (PC) remains a deadly malignant disease, with a 6% five-year survival rate, and increased incidence and mortality in recent years [63]. It has an extremely poor prognosis due to many factors, including low diagnosis rate, a high rate of metastasis, and the poor efficacy of conventional treatments [64].

Evidence showed that urinary matrix metalloproteases (uMMP-2) and urinary tissue inhibitor of metalloproteases (uTIMP-1) can be used to detect PC, and uTIMP-1 may be used to distinguish between pancreatic ductal adenocarcinoma (PDAC) and pancreatic neuroendocrine tumors [10]. Wang et al. reported that macrophage inhibitory cytokine 1 (MIC-1/GDF15) was overexpressed in PDAC tissues and may be a novel biomarker to screen for PDAC [11]. Moreover, Jiang et al. demonstrated that the expression of RGS6 was low in PC patients [12]. In general, the peptides mentioned above may serve as novel diagnostic biomarkers in PC.

Rothenberg et al. have identified that gemcitabine, as a first-line treatment, can improve the survival rate and quality of life in cases of advanced pancreatic cancer. However, the median survival and one-year survival rate were approximately 6 months and 18%, respectively [65]. In recent years, peptide-based vaccines, which elicit a specific anticancer response, have been considered to be a promising treatment option. Tumor-associated antigens (TAAs) can be recognized by the immune system and thus result in the disturbance of cancer cells or even tumor regression [66]. However, peptide vaccines showed limited clinical efficacy, influenced by the ability of tumor cells to escape recognition by the immune system [66]. Multiple mechanisms might contribute to immune escape such as a loss or downregulation of molecules, including tumor antigens and human leukocyte antigen (HLA) [67]. To date, some novel potential solutions or modulations have been proposed, such as the modification of TAA peptides, vaccines against multiple TAA epitopes, and the combination of chemotherapy [66].

Recently, the Wilms tumor gene (WT1) peptide-based vaccine in combination with gemcitabine was found to be more effective than gemcitabine alone. The median survival and one-year survival rate of the combination therapy were 8.1 months and 29%, respectively [27]. WT1 is overexpressed in PC cells, and the WT1 protein acts similarly to TAA and is targeted by specific effector T cells in immunotherapies. WT1-specific cytotoxic T lymphocytes (CTLs) against PC cells and delayed type hypersensitivity (DTH) were induced in response to the WT1 peptide-based vaccine. Through the release of perforins and granzymes as well as FasL/Fas interactions, the target tumor cells were eliminated and regressed. Moreover, CTLs specific for WT1 only act on cells with elevated expression of WT1 but do not damage normal cells, such as hematopoietic cells [68]. Therefore, the WT1 vaccine has no significantly adverse effects on hematopoiesis [68].

Gemcitabine induces cell apoptosis through inhibiting DNA synthesis [69]. The drug has many immune-modulating functions, such as the selective depletion of B lymphocytes, the reinforcement of T-cell recall responses, the reduction of regulatory T-cells, and an increase in the cross-presentation and cross-priming of tumor antigens [70–72]. Furthermore, gemcitabine upregulates the expression of WT1 and enhances the sensitivity of pancreatic cancer cells to CTL-mediated killing [64, 73].

WT1 peptide-based vaccines upregulate WT1-specific CTLs, and gemcitabine contributes to the amplification of CTL proliferation and the antitumor response. Thus, the combination of the WT1 vaccine with gemcitabine was synergistic [73]. Nishida et al. proved that longer survival was significantly interrelated with a positive DTH to WT1 peptides, and a high frequency of memory-phenotype WT1-specific CTLs was observed among DTH-positive patients [27]. Positive DTH to WT1 and a higher frequency of memory-phenotype WT1-CTLs could serve as two useful prognostic markers of effective clinical results [27]. In addition, the WT1 vaccine led to pain relief and alleviated distressing symptoms. The side effects of combination therapy resemble those of gemcitabine alone except for topical skin reactions [27]. The combination of chemotherapy with immunotherapy against cancer proved to be effective and synergistic.

In addition, Suzuki et al. reported that a KIF20A-derived peptide combined with gemcitabine increased the number of peptide-specific IFN-*γ* producing cells and indicated promising clinical outcomes in advanced PC patients [28]. Moreover, a mixture of a telomerase (GV1001) vaccine and gemcitabine was found to be safe, however, with a weak and transient immune response [29].

## 5. Peptides and Gastric Cancer

In spite of decreased incidence and death rates of gastric cancer worldwide in recent years, gastric cancer still has high incidence rates, especially in Eastern Asia, Eastern Europe, and South America [63]. The incidence of gastric carcinoma has notably decreased due to improved hygiene leading to lower rates of* H. pylori* infection, the popularization of refrigeration, and reduced smoking rates [74]. Chemotherapy, such as docetaxel, cisplatin, 5-fluorouracil, and S-1, is the conventional treatment for advanced, recurrent, or unresectable gastric carcinoma and shows poor clinical prognosis [75]. Since the acceptance of Provenge (sipuleucel-T) as the first cancer vaccine in prostate cancer by the FDA, peptide vaccine therapy was widely attempted in other cancers, such as colorectal, pancreatic, and gastric cancer [8].

Peptides are capable of detecting and diagnosing cancer. Zheng and his colleagues reported that leucine-rich repeat-containing G protein-coupled receptor 5 (LGR5) levels were significantly elevated in gastric cancer tissues, thus serving as an early diagnostic biomarker [13]. In addition, the detection of serum pepsinogen I (PGI), pepsinogen II (PGII), and carbohydrate antigen 242 (CA242) may be useful in diagnosing gastric cancer [14].

Recently, clinical trials in patients with gastric cancer (GC) have been conducted using peptide-based vaccines, including vascular endothelial growth factor receptor 2- (VEGFR2-) 169, VEGFR1-1084, and lymphocyte antigen 6 complex locus K (LY6K-177) epitope peptides [30, 31].

VEGF is highly expressed in endothelial cells of newly formed tumor vessels and is considered to be a tumor angiogenic and vasculogenic factor [76]. VEGFR2 is responsible for mitogenesis, angiogenesis, and permeability-enhancing activity by binding with VEGF, while VEGFR1 plays a negative role in VEGF-induced responses by inhibiting the binding of VEGFR2 with VEGF [77]. A peptide vaccine targeting VEGFR1 and an anti-VEGFR2 antibody are effective in inhibiting tumor angiogenesis [77, 78]. Therefore, VEGFR1 and VEGFR2 are promising antiangiogenic targets [79]. A combination of a VEGFR1 and VEGFR2 peptide-based vaccine with S-1 plus cisplatin showed improved clinical efficacy, and no severe adverse events were observed in patients with advanced GC. The median overall survival and progression-free survival time were 14.2 months and 9.6 months, respectively, with the combined therapy, compared to 13 months and 6 months with the S-1 plus cisplatin treatment [30].

LY6K-177 is overexpressed in the majority of lung and esophageal cancer tissues [80]. Ishikawa et al. have demonstrated that the peptide vaccine derived from the HLA-A^*∗*^2402-restricted LY6K-177 epitope was able to induce a specific CD8+ CTL response [81]. The suppression of LY6K expression with siRNA effectively inhibited the growth of LY6K-expressing lung and esophageal cancer cells [81]. Therefore, LY6K might be suitable to repress tumor growth as a targeting peptide in vaccine therapy. Clinical trials of vaccine therapy containing peptide LY6K-177 have verified that the LY6K-177 vaccine stimulated an antigen-specific CD8+ CTL response and significantly prolonged the survival of patients with esophageal squamous cell carcinoma [82]. An estimated 85% of GC patients have LY6K expression. A phase I clinical trial of an LY6K-177 peptide vaccine emulsified with Montanide ISA 51 was conducted in advanced gastric cancer patients [31]. The clinical response was effective, with nearly no side effects except for redness and induration at the injection sites [58].

To a great extent, the development of immunotherapy with peptide vaccines depends on the identification of novel vaccine targets, such as tumor-associated antigens [65]. However, few gastric cancer-targeting TAAs have been identified, and TAAs that can induce anticancer responses need to be further investigated.

## 6. Peptides and Prostate Cancer

Prostate cancer is the first leading cancer type of all newly diagnosed cancers and the second leading cause of cancer deaths among men in 2014 [63]. The incidence rate has declined but has fluctuated greatly since 2000 due to differences in prostate-specific antigen (PSA) testing prevalence and ethnicity [83]. A variety of chemotherapies have been employed to clinically treat prostate cancer, such as docetaxel and abiraterone [84]. Immunotherapy has been shown to be feasible to cope with chemotherapy-resistant cancer. Sipuleucel-T is the first FDA-approved cancer vaccine for the treatment of castration-resistant prostate cancer patients [85].

Mcgrath and his colleagues validated that EN2, a homeobox-containing transcription factor, was present in human fetuses but absent in healthy adults. However, the overexpression of EN2 in patients with prostate cancer can lead to the diagnosis of prostate tumors [15]. Similarly, mitochondrial uncoupling protein 2 is overexpressed in prostate cancer and may serve as a biomarker for diagnosis [16].

Faced with limited therapeutic efficacy, the identification of novel tumor antigens and the elevation of immunogenicity using vaccines are advisable approaches to improve clinical responses [66]. Noguchi et al. reported that personalized peptide vaccination (PPV) was well tolerated in the treatment of patients with castration-resistant prostate cancer (CRPC) in 2013 [47]. As we mentioned previously, although a large number of clinical trials have been conducted, outcomes showed limited responses and were less than satisfactory [86]. The limited clinical efficacy might be caused by not knowing the immunological status of patients, which contributed to mismatches between vaccine peptides and the heterogeneous immune cell repertoires [87].

Unlike other therapies, PPV is a novel immunotherapy tailored for individual patients. Many suitable candidate antigens are selected based on HLA type and the preexisting host immunity [88]. Conventional vaccines containing simply one peptide might not initiate a specific antitumor response against tumor cell variants because of the loss or reduction of TAA [88]. Therefore, a maximum of four peptides might increase the possibility of inducing immune responses and thus decrease the chance of tumor escape from immunosurveillance [89]. Two to four selected peptides were employed in patients during this trial along with incomplete Freund's adjuvant. The estimated median survival time was 18.8 months [47]. The result demonstrated that PPV was feasible for patients with CRPC and also recommended a surrogate marker for the evaluation of the clinical efficacy of cancer vaccine prostate-specific antigen doubling time (PSADT) [47].

In 2014, Saif reported that the PAP-114-128 epitope-based vaccine stimulated antigen-specific T-cell responses and reduced the growth of prostate cancer cells in C57BL/6 mice [32]. Prostatic acid phosphatase (PAP) is overexpressed in prostate cancer and may be an ideal vaccine target in the immunotherapy of prostate tumor patients [8]. Because long peptide vaccines are more efficient than ones that use whole proteins [90], the PAP-114-128 epitope peptide, which can induce CD4+ and CD8+ T cell responses, was screened from the PAP protein. The trial also validated that the PAP-114-128 peptide delivered through the ImmunoBody vector (IB-PAP-114-128) exhibited stronger CD4+ and CD8+ T cell specific responses and IFN-*γ* response than the PAP-114-128 peptide [32]. Furthermore, the IB-PAP-114-128 vaccine stimulated T cells that had higher avidity than PAP-114-128 emulsified with incomplete Freund's adjuvant [32]. ImmunoBody uses monoclonal IgG1 antibodies that were reconstructed to express specific antigenic epitopes to induce cellular immunity [91]. The Fc region of IgG1 can elicit high-affinity responses when targeting Fc*γ*R (CD64) expressed on DCs [92]. Thus, the fusion of the PAP-114-128 epitope peptide and ImmunoBody vector demonstrated effective antitumor benefits [32]. Further studies of this combined therapy are needed to assess its clinical efficacy.

Additionally, Fenoglio et al. confirmed the safety and immunological response against prostate tumors by using of a multipeptide, dual-adjuvant telomerase vaccine called GX301, which is composed of four telomerase peptides (peptide540–548, peptide611–626, peptide672–686, and peptide766–780) and two adjuvants, MontanideISA-51 and Imiquimod [48].

## 7. Peptides and Breast Cancer

Breast cancer is the second most common cause of cancer deaths among women in the United States in 2014 [63]. The increased incidence rates but decreased death rates of breast cancer might be attributed to the prevalence of screening examinations, early diagnosis that prevents tumors from developing into advanced stages and improvement in treatment [93, 94].

At present, treatments for breast cancer patients consist of chemotherapy, endocrine therapy, immunotherapy, and combination therapies [95]. Immunotherapies, including antibodies and peptide vaccines, are effective in the treatment of chemotherapy-resistant cancer [96]. Trastuzumab is a monoclonal antibody against human epidermal growth factor receptor 2 (HER-2) [97], which is overexpressed in almost 30% of breast cancer patients and is closely related to poor prognosis [17]. Ado-trastuzumab emtansine has been approved by the FDA as standard regimen for patients with HER-2 positive breast cancer [98].

The earlier the diagnosis occurs, the better the prognosis will be. Peptides play important roles in the early diagnosis of breast cancer, which results in decreased mortality. A list of antigens expressed in breast cancer cells including HER-2, carcinoembryonic antigen (CEA) mucin1 [18], p53, and telomerase reverse transcriptase has been investigated in humans [96, 99]. These peptides may be used to detect breast cancer.

In recent years, many strategies to improve immune efficacy have been proposed, such as the modification of peptide sequences at amino acid residues [66], using different vaccine delivery systems [95], PPV therapy as mentioned previously [87], and various combination therapies.

Takahashi et al. reported that personalized peptide vaccination applied clinically to metastatic recurrent triple-negative breast cancer (TNBC) patients has demonstrated feasible results [9]. The advantages of PPV over conventional immunotherapy methods have been reported previously. TNBCs, lacking the immunohistochemical expression of HER-2, the estrogen receptor, and the progesterone receptor, occur more frequently in younger women [100]. TNBC patients often have a poor prognosis and present with an aggressive grade and lymph node metastases at the time of diagnosis [101]. In this trial, most patients displayed augmented PPV-induced immune responses, showing considerable efficacy [9]. Moreover, no patient had severe therapy-related adverse events throughout the treatment [9].

According to previous studies, different vaccine delivery systems also greatly affect the clinical immune efficacy and demonstrated augmented immune responses [33]. Cationic liposome enhanced the amplitude of the antitumor effect and resulted in tumor regression when used as an adjuvant treatment [102]. There are many advantages that contribute to the adjuvant performance, such as versatility in lipid composition and size, the high efficiency of antigen loading, increased presentation of antigens, and the high ability of biodegradability and biocompatibility [103]. Mansourian et al. reported on a p5 peptide (HER-2 derived peptide) encapsulated in a delivery system that is composed of fusogenic dioleoyl phosphatidylethanolamine (DOPE) incorporated into 1,2-dioleoyl-3-trimethylammonium-propane (DOTAP) cationic liposome-cholesterol, and when the peptide was coadministered with CpG-ODN, the system increased the delivery of p5 and showed an elevated specific CTL response in mice inoculated with TUBO tumor cells [33]. TUBO is a cloned cell line that overexpresses the rat HER-2 protein [33]. The study demonstrated that further studies on its clinical effects in HER-2 positive breast cancer patients would be warranted. In addition, Shariat et al. investigated optimal methods of encapsulating the p5 peptide into liposomes to improve the peptide encapsulation efficiency [49]. Moreover, Karkada and colleagues designed a liposome-in-oil vaccine platform called DepoVax that can enhance immunogenicity of the vaccine [104]. Effective vaccine delivery systems play important roles in vaccine therapeutic efficacy.

Additionally, Ohtake and his colleagues validated that an artificial long peptide consisting of survivin-18 (SU18) and SU22 connected by a glycine linker was able to induce IFN-*γ* producing Th1 and Tc1 cells better than mixed short peptides [34]. In addition, Mittendorf et al. reported that the combination of E75 and granulocyte-macrophage colony-stimulating factor (GM-CSF) is safe and inhibits tumor recurrence [35]. Moreover, evidence has suggested that a multiepitope derived from an ErbB-2 vaccine suppressed the growth of breast cancer stem cells and consequently prevented tumorigenesis [36].

## 8. Discussion

Cancer is a threat to human health [105] due to its metastatic characteristic and high recurrence and mortality rates. Tumor cells can survive by escaping the host's immune system, synthesizing proteins to resist external treatment, and so forth [106, 107]. Traditional cancer treatments include chemotherapy, radiotherapy, and surgical resection. Among these treatments, chemotherapy remains a helpful and frequently used method to treat cancer. Commonly, traditional chemotherapeutical drugs target tumor cells by disrupting necessary cell products, such as DNA, RNA, or proteins [108]. However, chemotherapy is also insufficient. Because chemotherapy does not specifically target tumor cells, it causes many side effects in patients [109]. Additionally, multidrug resistance (MDR) is the main reason that chemotherapy fails to cure patients [108]. Under these limitations, chemotherapy based on peptides has received increased attention.

Peptides, which are short chains of amino acid monomers linked by peptide bonds, can specifically bind to tumor cells with low toxicity to normal tissues [110], indicating that they are a promising anticancer agent. This tumor-targeting ability of peptides is based on molecular structure [110]. Tumor cells have different membrane proteins on the cell membrane, such as endothelial cell growth factor receptors (EGFR) and cell surface proteoglycans [111, 112], making it possible for molecules to specifically bind to these proteins [113]. Peptides, derived from natural or synthetic sources, can selectively bind to these proteins [110] because they may share similar structures by containing arginine and lysine [114]. These amino acids can form hydrogen bonds with the negatively charged components on the cell membrane [114, 115], indicating that amino acids are the main reason why peptides may bind to tumor cell membranes. However, these properties are not sufficient for peptides to specifically target tumor cells. The specific selective ability of peptides may depend on their spatial structure [116], such as cartilage matrix proteins, which have a three-stranded a-helical coiled-coil structure in the C-terminal domain that may serve as a trimerization site [117]. Peptides are not the only molecule that can bind to tumor cell membranes, but they are the most ideal molecules because they have low molecular weights and good cellular uptake [110].

As we have summarized previously, peptides can be used to treat different types of cancer (lung cancer, CRC, pancreatic cancer, gastric cancer, prostate cancer, and breast cancer), from early diagnosis, treatment to prognosis. In addition to these types of cancer, peptides can also be used in skin cancer, renal cancer, osteosarcoma, and so forth. Wu et al. reported that properdistatin, a novel peptide derived from the plasma protein properdin, could inhibit angiogenesis in A-07 human melanoma xenografts [110]. Liu and Miao also showed that the CycMSH peptide conjugated with Tc-99m has exhibited an ability to target metastatic melanoma, indicating the potential of metastatic melanoma detection by CycMSH [118]. González et al. reported that the peptides derived from the melanocortin 1 receptor could elicit cytotoxic T-lymphocyte responses to kill melanoma cells [119]. Additionally, peptides for renal cancer treatment have also been of great interest. Vacas et al. reported that a vasoactive intestinal peptide inhibited invasion and metastasis of ccRCCs, by decreasing the nuclear level of *β*-catenin [120]. In addition, a peptide-based vaccine has also been used to treat metastatic renal cell carcinoma. Yoshimura et al. reported that vaccination with a vascular endothelial growth factor receptor 1 peptide showed anticancer effects in 18 patients with metastatic renal cancer [121]. Rausch and colleagues also found that a vaccine based on the IMA901 peptide could elicit a T-cell response and prolong overall survival in patients with metastatic renal cell carcinoma [122]. In addition to the novel applications of peptides in diagnosing and treating cancer, peptides can be used in other aspects of cancer therapy. Liu et al. found that the peptide Myr-NR2B9c could be used to reduce bone cancer pain, suggesting that this peptide could be used in patients with advanced bone cancer [123]. From these data, we conclude that peptides could be used in treating many types of cancer, and this treatment has shown promising clinical outcomes.

Peptide based chemotherapy is also a type of immunotherapy (Table 2). Though the immune system can target tumor cells, the tumor cells develop a number of immune escape mechanisms to avoid immune system surveillance [124]. The mechanism of peptides used in cancer therapy can be divided into two aspects: (1) peptides can bind to specific molecular targets on tumor cells, and these peptides can either regulate the biosynthesis of tumor cells or serve as a drug delivery system. (2) Peptides can induce specific T cell responses to tumor cells, as González et al. reported [119].

Interestingly, peptides could also target tumor vessel as well as targeting tumor cells. Li and Cho thought that tumor vascular was a better target for peptide treatment, when compared to tumor cells [125]. The endothelial cells of tumor have unique advantages in attracting peptide, such as low drug resistance, distinct microenvironment, and better blood perfusion [38, 126–128]. These advantages also give the unique application of peptide treatment, such as direct molecular imaging of targeted vascular peptides [125]. These peptides could provide potential target in diagnosing and treating tumor.

In using peptides to treat cancer, peptide-based vaccines have drawn increased attention (Table 3). Peptide-based vaccines have been widely applied in various diseases, such as allergies, infectious diseases, autoimmune diseases, and even cancer. Recently, peptide-based vaccines against cancer have been used to elicit tumor regression. Since the acceptance of sipuleucel-T by the FDA as the first peptide vaccine for prostate tumors, an increasing number of clinical trials have been conducted in many other cancer types, such as melanoma, glioblastoma, breast cancer, and gastric cancer [8]. A peptide-based vaccine has many advantages, including (1) the convenient and inexpensive acquisition of peptides; (2) easy administration; (3) the specificity of targeting to tumor tissues but not normal tissues; (4) fewer or even no severe side effects [66].

Tumor-associated antigens (TAAs) are expressed in tumor cells and can be recognized by T lymphocytes, resulting in activation of the immune system [66]. A TAA peptide vaccine, when injected into cancer patients, binds with the restricted major histocompatibility complex (MHC) molecule expressed in antigen presenting cells (APCs) [129]. Then the peptide/MHC complex is transported to the cell surface after intracellular processing and recognized by T cell receptor (TCR) on the surface of T cells, leading to the activation of T lymphocytes [130]. Therefore, a peptide cancer vaccine may elicit a specific immune response against tumors. Nevertheless, the clinical response is limited and shows limited efficacy [131]. These failures may be due to many factors, including the poor immunogenicity of TAAs, immune escape of tumor cells, and tumor heterogeneity [67]. New strategies for improving the clinical outcome include the modification of TAA peptides [132], vaccines against multiple TAA epitopes, personalized peptide vaccination [87], a combination with chemotherapy, and different administration routes and delivery systems [95]. Some novel methods have been tried, and some may improve the clinical efficacy and prolong the survival of cancer patients.

## 9. Conclusion

In this review, we mainly summarized new advances in using peptides to treat different types of cancer, indicating that peptides could be used as an ideal immunotherapy method in treating cancer due to the novel advantages of peptides, such as specifically targeting tumor cells, decreased toxicity and efficient immunoreaction. The development of identifying and synthesizing novel peptides could provide a promising choice to patients with cancer.

## Figures and Tables

**Table 1:** Peptides applied in cancer diagnosis.

Cancer	Peptide	Year	Author	Reference
Pancreatic cancer	uMMP-2 and uTIMP-1	2014	Roy et al.	[10]
	MIC-1/GDF15	2014	Wang et al.	[11]
	RGS6	2014	Jiang et al.	[12]

Gastric cancer	LGR5	2013	Zheng et al.	[13]
	PGI/II, CA242	2014	Lu et al.	[14]

Prostate cancer	EN2	2013	McGrath et al.	[15]
	UCP2	2013	Li et al.	[16]

Breast cancer	HER-2	2014	Boku	[17]
	MUC1	2011	Zanetti et al.	[18]

Colorectal cancer	HNP1-3	2006	Albrethsen et al.	[19]
	CPAA-783-EPPT1	2012	Bloch et al.	[20]
	Serum C-peptide	2014	Comstock et al.	[21]

Lung cancer	Linear peptide antigen derived from ANXA1	2014	Wang et al.	[22]
	C-peptide in serum	2014	Zhang et al.	[23]
	11 novel peptides	2014	McGuire et al.	[24]
	Peptides from variable parts of antibodies	2014	de Costa et al.	[25]
	HCBP-1	2014	Wang et al.	[26]

**Table 2:** Peptides applied in treating cancer.

Cancer type	Peptide	Model	Year	Author	Reference
Pancreatic cancer	WT1	In vivo	2014	Nishida et al.	[27]
	KIF20A	In vivo	2014	Suzuki et al.	[28]
	GV1001	In vivo	2014	Staff et al.	[29]

Gastric cancer	VEGFR1, 2	In vivo	2012	Masuzawa et al.	[30]
	LY6K-177	In vivo	2014	Ishikawa et al.	[31]

Prostate cancer	PAP-114-128	TRAMP C1	2014	Saif et al.	[32]

Breast cancer	p5	TUBO	2014	Mansourian et al.	[33]
	SU18, SU22	In vivo	2014	Ohtake et al.	[34]
	E75	In vivo	2014	Mittendorf et al.	[35]
	ErbB-2	MMC	2014	Gil et al.	[36]

Colorectal cancer	ANP	DHD/K12/Trb, SW620	2012	Serafino et al.	[37]
	TCP-1	HCT116 and HT-29	2010	Li et al.	[38]
	F56	HUVEC	2014	Wang et al.	[39]

NSCLC	WT1	In vivo	2013	Takahashi et al.	[40]
	TERT572Y	In vivo	2014	Kotsakis et al.	[41]
	Disruptin	H1975	2014	Ahsa et al.	[42]
	TREM-1	J774A.1	2014	Sigalov	[43]

Lung cancer	LFC131	A549	2014	Chittasupho et al.	[44]
	BPP	In vivo	2014	Wang et al.	[45]

Melanoma	TH10	B16F10-luc-G5	2014	Guan et al.	[46]

**Table 3:** Peptide-based vaccine in clinical application.

Cancer	Treatment	Sample	Study phase	Year	Author	Reference
Pancreatic cancer	WT1 peptide-based vaccine combined with gemcitabine	32	I	2014	Nishida et al.	[27]
	KIF20A-derived peptide in combination with gemcitabine	9	I	2014	Suzuki et al.	[28]
	Telomerase GV1001 vaccine together with gemcitabine	21	I	2014	Staff et al.	[29]

Gastric cancer	VEGFR1-1084 and VEGFR2-169 combined with S-1 and cisplatin	22	I/II	2012	Masuzawa et al.	[30]
	LY6K-177 peptide vaccine emulsified with Montanide ISA 51	6	I	2014	Ishikawa et al.	[31]

Prostate cancer	PPV (2-4 positive peptides selected from 31 candidate peptides)	100	II	2013	Noguchi et al.	[47]
	IB-PAP-114-128 vaccine therapy	—	—	2014	Saif et al.	[32]
	A multipeptide, dual-adjuvant telomerase vaccine (GX301)	11	I/II	2013	Fenoglio et al.	[48]

mrTNBC	PPV	18	II	2014	Takahashi et al.	[9]

Breast cancer	P5 encapsulated in DOTAP-cholesterol-DOPE liposomes coadministered with CpG-ODN	—	—	2014	Mansourian et al.	[33]
	Optimized encapsulation of p5 into liposomes	—	—	2014	Shariat et al.	[49]
	An artificial long peptide consisting of SU18 and SU22	—	—	2014	Ohtake et al.	[34]
	Combination therapy of E75 and GM-CSF	187	I/II	2014	Mittendorf et al.	[35]
	Multiepitope derived from ErbB-2 vaccine	—	—	2014	Gil et al.	[36]

Colorectal cancer	Atrial natriuretic peptide (ANP)	—	—	2012	Serafino et al.	[37]
	TCP-1 peptide	—	—	2010	Li et al.	[38]
	F56-VCR-NP	—	—	2014	Wang et al.	[39]
	Peptides from common bean NDF	—	—	2014	Luna Vital et al.	[50]
	Cep55/c10orf3 derived peptide vaccine	—	—	2011	Inoda et al.	[51]
	A cocktail vaccine of 5 peptides	18	I	2014	Hazama et al.	[52]
	A combination of 7-peptide cocktail vaccine and tegafur-uracil plus leucovorin	30	I	2014	Okuno et al.	[53]

Lung cancer	Dendritic cell vaccines pulsed with WT-1 peptide	62	—	2013	Takahashi et al.	[40]
	hTERT-targeting Vx-001 vaccine	46	II	2014	Kotsakis et al.	[41]
	A synthesized peptide Disruptin	—	—	2014	Ahsa et al.	[42]
	A ligand-independent peptide-based TREM1 inhibitor	—	—	2014	Sigalov	[43]
	Peptide fractions from high oleic acid soybean	—	—	2013	Rayaprolu et al.	[54]
	Peptides from venom of Eastern green mamba	—	—	2014	Conlon et al.	[55]
	LFC131-DOX NPs delivery system	—	—	2014	Chittasupho et al.	[44]
	TH10-DTX-NP	—	—	2014	Guan et al.	[46]
	BPP-decorated chitosan nanoparticles	—	—	2014	Wang et al.	[45]

## Abbreviation

FDA: Food and Drug Administration
CRC: Colorectal cancer
CPAA: Cationic polyacrylamide
NDF: Nondigestible fractions
NSCLC: Non-small-cell lung cancer
PC: Pancreatic cancer
HER-2: Human epidermal growth factor receptor 2
CEA: Carcinoembryonic antigen
TAA: Tumor-associated antigens
GC: Gastric cancer
HLA: Human leukocyte antigen.
